# Feasibility and adherence to ecological momentary assessment among community-dwelling adults with suicide risk

**DOI:** 10.1186/s12912-025-03432-y

**Published:** 2025-07-01

**Authors:** Hyein Kim, Seongae Kwon, Sunyoung Park, Chaehyeon Kang, Heejung Kim

**Affiliations:** 1 Nell Hodgson Woodruff School of Nursing, Emory University, Atlanta, GA USA; 2 Brain Korea 21 FOUR Project, Yonsei University College of Nursing, 50-1 Yonsei-ro, Seodaemun-gu, Seoul, 03722 Republic of Korea; 3 College of Nursing, Yonsei University, Seoul, South Korea; 4 Department of Psychiatry, National Health Insurance Service Ilsan Hospital, Gyeonggi, South Korea; 5 Mo-Im Kim Nursing Research Institute, Yonsei University, Seoul, South Korea; 6 Institute for Innovation in Digital Healthcare, Yonsei University, Seoul, Republic of Korea

**Keywords:** Suicide prevention, Suicidal ideation, Suicidal impulse, Telemedicine, Feasibility studies, Psychiatric nursing, Community health nursing

## Abstract

**Background:**

Ecological momentary assessment (EMA) has seen increasing application in mental health research. However, there is a challenge in applying EMA to assess daily suicide risk in community settings due to poor adherence to the complex protocol and high dropout rates. The aim of this study is to assess the feasibility and adherence to the EMA when monitoring the daily risk of suicide in community-dwelling adults with suicidal ideation.

**Methods:**

This secondary analysis was based on primary data from an observational study. The study participants with suicidal ideation responded to a 28-day EMA online survey and pressed an event marker on an actigraphic device when feeling strong suicidal impulses. Feasibility was evaluated using the EMA response rate and actigraphic device adherence rate based on descriptive statistics. Mental health characteristics related to feasibility were assessed in self-reporting questionnaires, and nonparametric correlation coefficients were identified to assess the relevance to feasibility.

**Results:**

A total of 22 participants were enrolled, with 20 remaining in the final sample (90.9%). The average EMA response rate was 82.05%, decreasing from 86.96% during the first 2 weeks to 76.31% in the second 2 weeks. The Actiwatch adherence rate was maintained at 98.1%. Actiwatch adherence and EMA response rates were moderately correlated (r =.53, *p* =.016). Higher depression and anxiety scores were associated with lower Actiwatch adherence, whereas a higher perceived stress score was associated with lower EMA response rates. The peak of suicidal impulse patterns in event button activations usually occurred between 9 to 10 pm, while activations were lowest in the early morning hours, particularly between 4 and 6 am.

**Discussion:**

This study indicated that EMA using smartphones and actigraphic devices were feasible to monitor suicidal ideation and impulse for a month in community-dwelling adults; thus, it could be a complementary tool to assess daily suicide risk. However, there are still challenges to be overcome when EMA-based monitoring in the community is used for those with mental vulnerability. Thus, mental health professionals should carefully tailor the pros and cons of EMA based on our findings to enhance this vulnerable group’s participation and adherence to EMA for suicide prevention.

## Manuscript

### Background

Suicide is the most challenging mental health issue worldwide. It is one of the leading causes of death, reaching more than 700,000 annual deaths globally [1]. To prevent suicide, current research suggests assessment guidelines for suicide risk factors or warning signs such as mental disorders (e.g., depression), prior suicidal attempts, and stressful life events based on history taking [1, 2]. In addition, understanding timely suicide risk assessment is critical because suicidal ideation has the characteristics of rapid onset and short duration [3, 4]. However, there is a significant time gap between the occurrence and reporting of suicidal ideation. The time gap and recall bias usually occur when suicidal ideation is assessed in traditional research methodology [3, 4]. Thus, it is necessary to develop better measurements so that the researcher can evaluate real-time suicidal ideation, impulses, or behaviors for timely intervention.

Ecological momentary assessment (EMA) is a prominent method for capturing real-time data in natural environments. Through EMA, people can continually report their moods and behaviors with short intervals of follow-up periods; therefore, individual self-reporting and recall bias can be minimized due to multiple reports in a timely manner [3]. Given that individuals with suicidal ideation have various fluctuations in daily moods and mental health risk factors, applying EMA is suitable for identifying the suicide risk of these individuals in daily life [4]. In the past decade, EMA methods have been widely used to assess individuals with suicidal ideation to overcome the limitations of primary assessment tools in mental health practice such as face-to-face interviews and self-reporting questionnaires [4–8].

However, there are some challenges to apply EMA of suicide risk in the clinical practice. First, the feasibility of EMA has been reported with wide ranges when documenting suicidal ideation and relevant risk monitoring [9–11]. A recent systematic review conducted by Kivelä and colleagues [9] reported that the utilization of EMA in suicide research was generally acceptable, but the acceptance rates widely ranged from 25% to 93%. Second, the compliance rate exhibited no significant variation between clinical or nonclinical populations, indicating that demographic variables did not exert a substantial influence on it. Instead, Forkmann et al. [10] reported that better compliance of EMA-based risk evaluation is depending on severity of symptoms, such as passive and active suicidal ideation and its proximal risk factors in depressed inpatients. Third, there is some discrepancy between paper-to-pencil versus Information and Communication Technology (ICT)-based measures. For example, Torous et al. [12] revealed that suicidal ideation can be detected more accurately using a smartphone application (hereafter, “app”) than a traditional paper-based experienced sampling method via the questionnaires. No participant reported suicidal ideation above level 2 based on the paper-based Patient Health Questionnaire-9 (PHQ-9) scale, whereas 69.0% (9/13) reported suicidality with this level using the EMA app.

Thus, it is important to enhance acceptability and compliance based on feasibility research when using multi-device EMA to monitor suicide risk. When using diverse measurement of EMA, it is likely to reduce measurement errors [3, 13]. For example, EMA captures active data such as mood and behavior in real-time settings, while actigraphy collects passive sensor data through continuous movement tracking [14, 15]. Actigraphy is a non-invasive method for objectively measuring sleep patterns, daytime activity levels, and physiological states using accelerometers embedded in wearable devices [16]. In the context of suicide risk monitoring, actigraphy provides additional information to record physiological and behavioral changes that may indicate proxy measures in suicidal ideation or impulse [17, 18]. Compared to active user engagement through self-reporting survey, actigraphy continuously records data without participant’s manual input, making it particularly valuable for assessing individuals who may not consistently report their mood or behaviors, especially mentally vulnerable patient groups [19].

To consider the current expansion of ICT-based EMA, it is crucial to examine the specific group’s feasibility to develop tailored strategies targeting a specific group’s health concern. In the field of ICT-based research, feasibility studies play a significant role in obtaining insights about expected problems and solutions, evaluating the practicality of conducting the primary studies, and refining the study protocols prior to the main study [20]. Moreover, this study focuses on those with suicidal ideation residing in the community, as they are hard-to-reach and vulnerable groups in ICT-based mental health care. Several challenges have existed when implementing EMA in community-based adults to evaluate their suicidal ideation. Rogers [11] reported that each participant felt burdened because of the intensive frequency of assessment. Forkmann et al. [10] found that compliance rates decreased over the survey period because of subject burden and reports of fatigue with the intensive assessment. Previous studies have used random or fixed survey schedules that did not account for each participant’s preference [10, 11]. To enhance adherence to EMA, it is vital to consider individual uniqueness and preferences to overcome the identified limitation during the EMA survey. When the data are more complete, it becomes easier to detect health problems. In mental health research, community-dwelling adults with suicide risk are a hard-to-reach population to be recruited for research because of their vulnerability and safety issues.

Thus, this study aimed to identify factors that relate to compliance with EMA to improve its implementation in suicide research. Specifically, this study focused on understanding how EMA can be optimized for assessing daily mood, suicidal ideation, and impulses through online surveys and actigraphy among community-dwelling adults with suicide risk. Specifically, this study proposed to (a) assess participant retention rate and adherence to EMA surveys and Actiwatch protocols, (b) identify factors related to the study adherence, and (c) describe the frequency of suicidal impulses in a day.

### Methods

#### Study design

This secondary analysis was based on primary data from an observational study [21, 22]. This retrospective exploration focused on evaluating the feasibility of and adherence to EMA and Actiwatch usage among community-dwelling adults at risk of suicide. In this secondary analysis, we assessed the feasibility of and adherence to these tools, adhering strictly to the published study protocol [21].

#### Participants

Participants were recruited with convenience sampling at a suicide-prevention center in South Korea. Eligibility criteria were as follows: (a) aged over 19 years; (b) attending a suicide prevention center as an outpatient; (c) owns a personal smartphone; (d) able to wear Actiwatch (Phillips Respironics, USA); (e) able to speak and write in Korean; (f) previous experience reporting suicidal ideation at least once based on the Korean version of the Beck Scale for Suicidal Ideation [23]; and (g) consents to participate. The original Beck Scale for Suicidal Ideation did not provide any specific cutoff score determining suicide risk [24]. The measurement has screening questionnaires for evaluating the presence of active or passive suicidal ideation. Therefore, participants in this study were characterized as community-dwelling people with current suicide risk if they reported any active or passive suicidal ideation. The exclusion criteria were as follows: (a) difficulty participating due to cognitive dysfunction, as determined by a psychiatrist; (b) moderately severe cognitive impairment at 65 years old at least; (c) difficulty participating due to active psychotic symptoms (i.e., auditory hallucination or delusion) that required hospitalization and urgent medication; and (d) current participation in another study. For this study, we determined a sample size of 20 participants to assess the feasibility and acceptability of EMA among community-dwelling adults. Pilot studies often include 10–30 participants to identify potential issues such as recruitment challenges, participant burden, and data collection difficulties before proceeding to larger trials [25]. Since pilot studies do not involve hypothesis testing, there is no fixed ‘rule of thumb’ for the exact number of participants; instead, the focus is on ensuring a sufficient number to detect major feasibility issues [26]. A sample of 20 was determined to be adequate to evaluate participant responses to frequent EMA prompts, assess adherence, and determine acceptability, thereby informing improvements for future studies.

#### Data collection

Details of data collection in the primary study protocol were reported [21]. In summary, the data were collected through (a) an EMA online survey conducted three times a day for 4 weeks (Days 1–28), (b) actigraphic data obtained by Actiwatch for first 2 weeks (Days 1–14), and (c) structured self-report questionnaires at baseline, the end of Week 2, and the end of Week 4. For this secondary data analysis, 28-day EMA reports, 14-day Actigraphy data, and survey data were used at baseline.

The feasibility assessment encompassed several key criteria: (a) participant retention; (b) adherence to EMA surveys and Actiwatch wearing time; and (c) activation of Actiwatch’s event marker. Participant engagement was quantified by calculating the percentage based on the number of participants initially enrolled after screening the eligibility and the dropout at the end of 4 weeks. EMA survey adherence was evaluated by calculating the ratio of the prompt sent by the research team to the participants versus the actual responses recorded in the online survey. Actiwatch adherence was determined by dividing the net duration for which participants wore the device by the total prescribed wear time. Furthermore, this feasibility paper investigated the predominant instances of suicidal impulse during the study period. Suicidal impulse was defined as very strong thoughts or impulses where they felt “I want to die now (In Korean: 나는 지금 죽고 싶어요)” or “I strongly want to attempt suicide now(In Korean: 나는 지금 매우 자살하고 싶어요).”

##### Ecological momentary assessment (EMA)

We collected real-time data of the participants’ mental health conditions, such as levels of depression, anxiety, and stress, as well as suicidal ideation, using an online survey platform. Each condition was assessed on a 5-point Likert scale (1 = none to 5 = very severe). Participants were able to report each condition three times a day through an individualized schedule. At baseline, participants gave us information about their usual time for waking up, going to bed, and feeling the suicidal impulse in a day. If they had strong suicidal impulses during the day at wake-up time or bedtime, the survey link prompt was set to be delivered only twice a day (i.e., wake-up time and bedtime). In addition, they could report “suicidal impulse” multiple times in a day as needed. Thus, the study participant received the individualized prompt via a text message containing the online survey link. The data were verified with the log of an online survey link and Actiwatch activation, especially for suicidal impulse.

##### Actiwatch

The levels of daytime activity, sleep pattern at night, and individual suicidal impulse were recorded via the wearable device Actiwatch Spectrum PRO (Philips Respironics, Pennsylvania, USA). It was required that participants always wore the Actiwatch, particularly on the nondominant wrist, except when bathing or engaging in water sports, such as swimming. This device collected data within a 15-second epoch time, with no need to charge 14 days during the data collection process. All the collected data were stored, deidentified, and saved via the Actiwatch Spectrum PRO program on a computer desktop. When suicidal impulses occurred, the participants pushed the event marker button. Given that Actiwatch is a wearable device and that pushing the button is more accessible than the EMA survey, they were encouraged to track their strong suicidal impulse while responding to the EMA survey three times a day.

##### Questionnaires

Participants’ mental health characteristics were assessed using structured self-report questionnaires at Weeks 0, 2, and 4. The Korean version of the Beck Scale for Suicidal Ideation [23] was also administered to evaluate whether the participants had current suicidal ideation. Other questionnaires used were the PHQ-9 (Korean version) [27], the Generalized Anxiety Disorder-7 (GAD-7) [28], the Perceived Stress Scale (PSS, Korean version) [29], and the Alcohol Use Disorder Identification Test-Korean (AUDIT-K) [30, 31]. The PHQ-9 [27] was used for measuring baseline depression, the GAD-7 [28] for baseline anxiety, and the PSS [29] for baseline stress level. Each of these measures has demonstrated strong psychometric properties in the published protocol paper [21].

#### Data extraction and analysis

Data analyses such as descriptive statistics, including frequencies with percentage, means with standard deviations [SD], Mann–Whitney U test, and Spearman correlation analysis, were conducted using IBM SPSS 26.0, with a significance level of α =.05. Given the small sample size (N = 20), Spearman correlation analysis was used to examine the correlation between EMA adherence and Actiwatch adherence. Furthermore, we computed the correlations between the EMA adherence rate, Actiwatch adherence rate, and baseline mental characteristics measured by PHQ-9 [27], GAD-7 [28], and PSS [29].

Responses stacked to the Google survey server were used to measure EMA survey adherence. The EMA response rates were capped at a maximum of 100%. Because it was mandatory for them to complete their reports three times a day to comply with our study protocol, the percentage was calculated based on three times a day for 28 days for each participant. Two research assistants downloaded the raw EMA data file via the Google survey server and screened them to evaluate redundant answers. The research team made an optimal rule for validating the authentic answers according to a 5-second rule, which is the rule for determining the accurate responses from the same answers. Once the participants provided duplicate answers within 5 seconds, the research team only selected the latest answers for the analysis. Considering the technical issues and the possibility that the participants’ indecisiveness would lead to redundant answers, the final EMA data were evaluated during group consultation with the principal investigator.

The Actiwatch adherence rate was calculated by dividing the total wearing time in minutes by the net wearing time of Actiwatch. The net wearing time of Actiwatch was calculated by subtracting the excluded wearing time of Actiwatch from the daily Actiwatch wearing time. The most suicidal impulse time was yielded according to the Actiwatch event marker frequency data using a bar chart.

#### Ethical considerations

This secondary data analysis was exempt from approval from the Institutional Review Board of the affiliated university (IRB No.4–2023-0096) in March 2023. The primary data were deidentified to protect participants’ confidentiality. Regarding safety, participants were permitted to call the research team after making suicidal attempts, to report difficulties in managing depression symptoms, and to request help. Participants were provided the contact information of 24/7 suicide hotlines and crisis lines at their baseline visits; these cases were handled by a national agency, and issues were reported to the related suicide-prevention center for further assistance. Although we collected real-time data via momentary assessments, our study did not guarantee active participant engagement or provide immediate interventions. Instead, we gathered retrospective information about past suicidal impulses during midpoint and endpoint visits. If a review of the previous 14 days of EMA data indicated a high risk, such as elevated suicidal ideation scores or any suicidal attempts, the research team promptly notified the participant’s healthcare provider. Likewise, if participants verbally reported suicidal thoughts or attempts, we again informed their providers to ensure appropriate follow-up. During the primary study, two participants had undergone emergency visits for suicidal attempts, and a post hoc report of suicidal attempts at the midpoint and endpoint of the survey was made. After obtaining confirmation, the research team referred them to the case managers of the suicide-prevention center or the 24/7 government suicidal center.

### Results

#### Sample characteristics

This study enrolled 23 participants who were being referred to the suicide prevention center from May to December of 2021. Among them, 20 remained for the final sample (Table 1). Their mean age was 27.79 ± 11.33 years, and most of them were women (n = 14; 70.0%). Table 1 presents their sociodemographic information. The participants were mostly high school graduates (n = 13, 65.0%) and single (n = 17, 85.0%). More than half of them perceived their health status as poor and did regular exercise (n = 12, 60.0%). According to AUDIT-K, the majority were at risk of alcohol problems (n = 15, 75.0%). Most of the participants had lifetime suicide attempts (n = 19, 95.0%) and were diagnosed with psychiatric disorders (n = 17; 85.0%). The most common disorder was depression, followed by other mood disorders (Table 2). In addition, most of them reported significant limitations in daily life because of the psychiatric symptoms. However, there were no differences in EMA and Actiwatch adherence depending on clinical characteristics (Table 2).

**Table 1:** Participants’ sociodemographic characteristics (*N* = 20)

	**M ± SD** or **n (%)**
**Age**	27.78 ± 11.33
**Sex**	
Female	14 (70)
Male	6 (30)
**Education level**	
Middle school	2 (10)
High school	13 (65)
College or above	5 (25)
**Marital status**	
Single	17 (85)
Married	1 (5)
Divorced/widowed	2 (10)
**Perceived health status**	
Good	2 (10)
Moderate	7 (35)
Poor	11 (55)
**Regular exercise**	
Yes	12 (60)
No	8 (40)
**Smoking**	
Current smoker	7 (35)
Not a current smoker	13 (65)
**Drinking classification based on AUDIT-K**	
Normal drinking	5 (25)
Hazardous drinking	5 (25)
Alcohol use disorder	10 (50)

*AUDIT-K* Alcohol Use Disorder Identification Test-Korean

**Table 2:** Suicide-related characteristics and mean differences tested by Mann–Whitney U test (*N* = 20)

Variables	n (%)	Actiwatch adherence rate (%, M ± SD)	P value	EMA response rate (%, M ± SD)	P value
**Lifetime suicide attempt**					
Yes	17 (85)	98.31 ± 2.16	.258	83.96 ± 20.28	.921
No	3 (15)	96.90 ± 3.71		84.12 ± 15.07	
**Diagnosis of psychiatric disorders**					
Yes	19 (95)	98.39 ± 2.07	.100	84.84 ± 19.38	.500
No	1 (5)	92.70		67.86	
**Limitation in daily life due to psychiatric symptoms**					
Yes	15 (75)	98.13 ± 2.39	>.999	85.56 ± 19.41	.612
No	5 (25)	98.01 ± 2.61		79.29 ± 20.07	
**Psychiatric disorders**^**+**^		NA		NA	
Depression	14 (29.2)				
Bipolar disorder	7 (14.6)				
Anxiety disorder	5 (10.4)				
Sleep disorder	5 (10.4)				
Alcoholism	3 (6.2)				
Panic disorder	3 (6.2)				
Bulimia nervosa	2 (4.2)				
Adult attention-deficit hyperactivity disorder	1 (2.1)				
Impulsive control disorder	1 (2.1)				
Obsessive–compulsive disorder	1 (2.1)				
Paranoid personality disorder	1 (2.1)				
Schizophrenia	1 (2.1)				
Personality disorder	1 (2.1)				
Post-traumatic stress disorder	1 (2.1)				
N/A	2 (4.2)				

*EMA* Ecological Momentary Assessment; *NA* not applicable

*Note:* Multiple responses were allowed; mean differences were tested by Mann–Whitney *U* test

**Table 3:** Correlation coefficients among variables (*N* = 20)

Variables	1	2	3	4
1. EMA response rate	1			
2. Actiwatch adherence rate	.53^*^	1		
3. Baseline PHQ-9	.40	−.64^**^	1	
4. Baseline GAD-7	−.14	−.47^*^	.75^**^	1
5. Baseline PSS	−.59^**^	−.28	.52^*^	.44

*EMA* ecological momentary assessment; *PHQ-9* Patient Health Quessionaire-9; *GAD-7* Generalized Anxiety Disorder-7; *PSS* Perceived Stress Scale

^*^p < 0.05, ^**^p < 0.01

#### Feasibility outcomes

##### Participant retention

Recruitment occurred from May to October of 2021. A total of 23 participants were recruited, but one participant was screened out because of the age criteria. Two participants withdrew from the study because of psychiatric hospital readmissions and time conflicts with new employment. Thus, 20 participants out of the eligible 22 were finally retained for 28 days (90.9%).

#### Adherence to EMA survey and actiwatch use

**Fig. 1:**
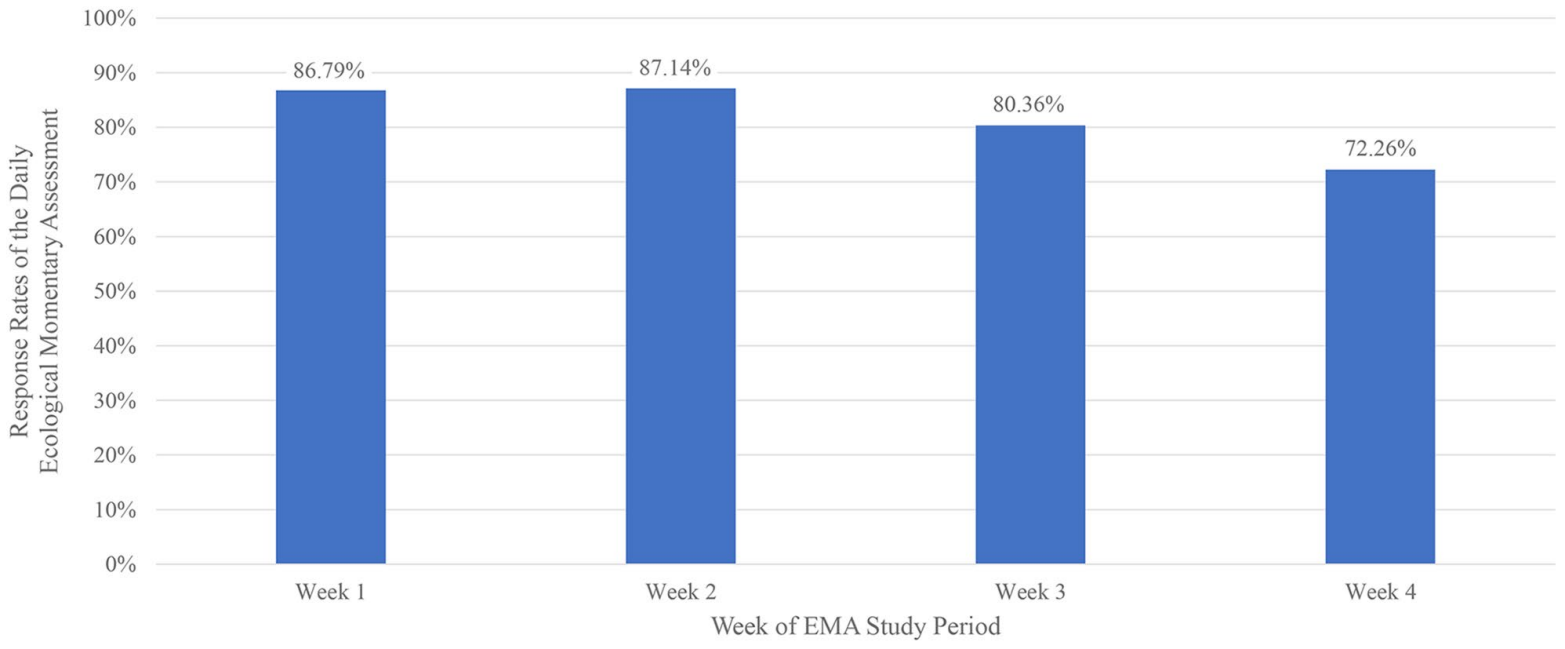
Response rates of the daily ecological momentary assessment

**Fig. 2:**
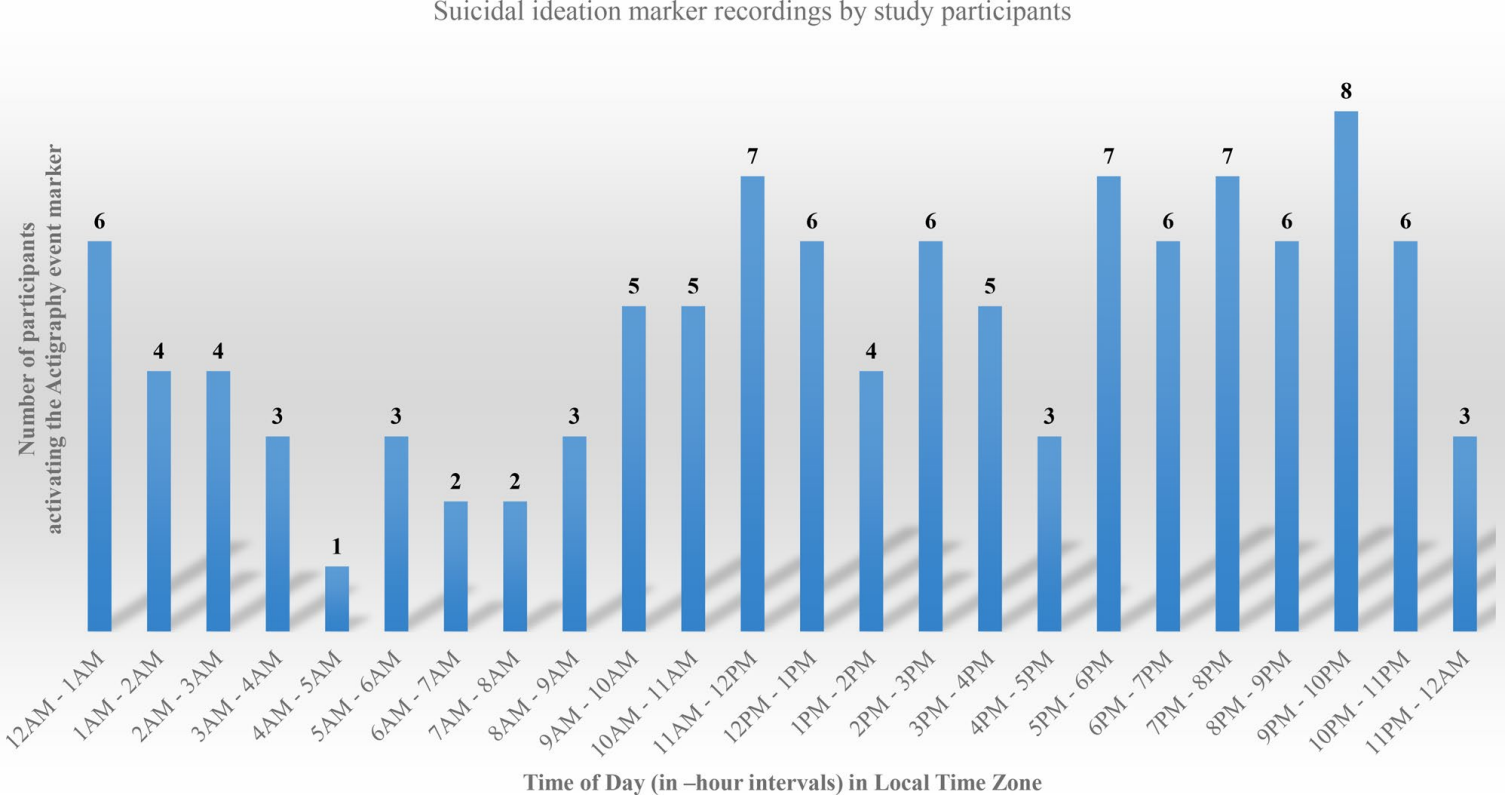
Recordings of suicidal ideation marker collected via Actiwatch

The average response rate for the EMA survey was 82.1% (range: 41.7–100%) among three times a day for 28 days. At the first half of the observational period (Days 1–14), the average response rate was 87.0%, but during the rest of the period (Days 15–28), it decreased to 76.3% (Fig. 1). Because the study participants wore the Actiwatch in the first 2 weeks (Days 1 to 14), the average Actiwatch adherence rate was 98.1% (13.68 out of 13.95 days). There were some discrepancies among the participants. On average, 82.05 ± 17.46% of EMA was reported per person. The average activation of the actigraphy button was 11.10 ± 14.40, ranging from 0 to 54. There were high discrepancies among the participants. Five participants never activated the button at all; however, eight individuals activated it more than ten times among 28 days. Figure 2 illustrates the number of participants activating the actigraphy button during each hour of the day. The data shows distinct suicidal impulse patterns, with a peak in button activations observed between 9 to 10 pm. A second increase was noted during midday (11am–12pm) and afternoon (5–6pm and 7–8pm), while activations were lowest in the early morning hours, particularly between 4 am and 6 am.

#### Factors related to EMA and actiwatch adherence

The Actiwatch adherence or EMA response rate showed no significant mean differences, depending on suicide-related characteristics, such as lifetime suicide attempts, diagnosis with psychiatric disorders, and significant limitations in daily life due to the psychiatric symptoms (Table 2). Table 3 showed a medium correlation between the Actiwatch adherence rate and EMA response rate (r =.53, *p* =.016). Participants who were more likely to respond to the EMA questions were more likely to adhere to wearing the Actiwatch. Furthermore, the baseline scores of PHQ-9 and GAD-7 were negatively associated with the Actiwatch adherence rate (PHQ-9: r = −.67, *p* =.003; GAD-7: r = −.44, *p* =.037), whereas the baseline PSS score was negatively associated with the EMA response rate (r = −.59, *p* =.007). Thus, the Actiwatch adherence rate was lower in the more depressed and anxious group, whereas the EMA response rate was lower in the more stressed group.

### Discussion

This study evaluated the feasibility of EMA for assessing daily suicide risk via an online survey in conjunction with Actiwatch usage among community-dwelling adults with suicidal ideation. Our study findings indicated a feasible acceptance rate of this combined approach. Notably, the study demonstrated a positive correlation between Actiwatch adherence and EMA response rates. Moreover, the mental health status of the participants was found to be linked to the overall rate of engagement in the study. The research stands out for its careful exploration of acceptance rates among the participants, considering the varying degrees of burden experienced by individuals based on the severity of their mental health status. The study findings regarding EMA response rates and Actiwatch adherence indicate the potential for voluntary assessment of suicidal ideation in the community settings that is open underreporting. Monitoring their responses and promptly notifying the appropriate mental health care provider during critical moments of risk might address their suicide risk.

This study demonstrated that EMA is feasible for adults with suicidal ideation living in the community, with a moderately high EMA response rate (84.0%) and a high Actiwatch adherence rate (98.1%). This EMA response rate was comparable to or higher than the rate obtained in similar studies, including 78% adults with major depressive disorder [12], 73% with major depressive disorder [32], and 69% at risk of suicide living in the community [11]. Furthermore, the weekly EMA response rate decreased over time, consistent with previous suicide studies using EMA [5, 33]. The decline in this rate occurred because of the subjects’ burden and fatigue caused by the intense assessment consistent to the previous study [5, 10]. This result suggests that fewer prompts to ask EMA questions can increase response and adherence rates. Nonetheless, our study participants showed an increase in EMA adherence after our midpoint survey because we had to meet them during this time. Although longer questionnaires were associated with a higher momentary burden than shorter versions [34], our well-developed EMA of 5-point Likert-type questions could alleviate the burden of 28-day momentary assessments.

Moreover, the Actiwatch adherence rate (98.1%) was higher than that of the previous study conducted in psychiatric adolescents, which showed a wearing rate of 76.1% (21.3 out of 28 days) [5]. This result could be related to the participants’ increased interest in this device, as shown in the sleep results obtained after wearing the device before participating in the study. A previous study used a different wrist-worn device (Empatica E4) in which the event marker was also pressed to measure distressed feelings; the average adherence rate was 9.74 days per participants and 95.3% in all days [6]; thus, it was similar to our results. Given that the adherence rate of wearable devices detecting suicidal ideation is still seldom reported, our study showed that the adherence rate for wearable devices possibly increased by meeting the needs of the participants during the study. Specifically, we customized the timing of the EMA survey prompts based on each participant’s baseline information, including their usual wake-up time, bedtime, and the time of day they most frequently experienced suicidal ideation. For participants who reported experiencing suicidal ideation primarily during specific times of the day, such as wake-up time or bedtime, the prompts were adjusted to twice daily at those specific times. This individualized approach ensured that the prompts aligned with participants’ daily routines and preferences, reducing the burden of participation and improving adherence rates for both the EMA surveys and Actiwatch wearing time.

The observed suicidal impulse trends in Fig. 2 emphasize the importance of tailoring EMA to participant behavior. Increased button activations during the evening and late-night hours (9–10 pm) suggest higher engagement and potential periods of vulnerability, whereas lower activations in early morning (4–6 am) may reflect decreased activity or sleep. These findings align with the circadian patterns of depression, which often intensify in the evening [35]. Symptoms of major depressive disorder show diurnal variations, with some patients experiencing more symptoms in evening [36]. Delayed biological rhythms or an evening chronotype are observed in individuals with major depressive disorder, and the degree of circadian rhythm misalignment is shown to correlate with symptom severity [37, 38]. Therefore, scheduling EMA prompts during peak engagement times, such as late evening, could improve adherence and enable early detection of suicidal impulses. Elevated evening activations may indicate critical windows for timely interventions, supporting the utility of wearable devices in identifying behavioral patterns and optimizing intervention timing in high-risk groups. This highlights the transformative potential of wearable technologies in suicide-prevention research. However, further studies with larger and more diverse populations are needed to confirm these findings and should explore these temporal patterns to enhance intervention precision and evaluate the broader applicability of wearable technology in suicide prevention.

Mental health conditions, including depression, anxiety, and stress levels measured by structured questionnaires, are negatively correlated with the EMA response rates and Actiwatch adherence rates. Thus, the mental health conditions at baseline were related to the reliability of the study design. This study also showed a gradually decreasing EMA response rate for 28 days of assessment. Participants with severe mental health problems are regarded as “hard to engage” in the study [39]. Relationships with the service providers and feelings of connectedness with suicide-related mobile apps are crucial factors for maintaining participants’ engagement in mental health services [39, 40]. Thus, those with mental health problems must be given access to the research team and clinical resources to improve their participation in the study. Future studies should also consider two points: (a) participants with severe mental health disorders or unmanageable mood symptoms have a greater burden to fully participate in the study than those with a relatively moderate degree of symptoms; and (b) decreased response should be carefully monitored during the study participation.

In this study, we observed participants’ irregular lifestyles as significant variability in participants’ daily routines, particularly in inconsistent sleep and wake patterns where they do not sleep at night and wake up in the morning, often aligning with circadian rhythm disturbances. This irregularity is commonly observed in individuals with a high risk of suicide [38]. To address this, we collected baseline data on participants’ usual routines and personalized EMA survey schedules accordingly. This approach was intended to minimize the impact of lifestyle irregularities on study adherence while accommodating participants’ unique needs and circumstances. The responses are difficult to validate when they report repeated responses in a short period of time. Hence, we set our own 5-second rule, which saved the very last response when multiple responses were recorded within 5 seconds based on our clinical experience interviewing psychiatric patients. In addition, the EMA response in one participant was intentionally ignored by the prompt. In this case, the response was considered to reflect the participants’ willingness to report and regard as their valid responses [40, 41]. Each response was labeled as wake-up time, most suicidal time, and bedtime; thus, even if recall bias was suspected, the response record was considered valid, given their sleep irregularity [42]. Our strategy can be utilized in similar future research that considers the participants’ circadian rhythms for the EMA study.

Accumulated data from Actiwatch event markers showed that suicidal impulse patterns can be possibly predicted. Figure 2 shows that the most suicidal impulse time can be inferred with EMA, considering that Actiwatch marker records were not based on prompts but rather on the participants’ willingness. In a previous study using Actiwatch for measuring sleep characteristics objectively, event markers were usually guided to be pressed when closest to sleep or wake onset [40]. In our study, we guided the participants to press the event marker when they had suicidal thoughts, consistent with the study by Kleiman et al. [6], which recorded suicidal thoughts on the wearable device Empatica E4 for adolescent inpatients with suicidal ideation. This study is also consistent with another suicide research that employs wearable or smart devices to assess the high-risk group’s suicidal thoughts and behaviors [5, 6]. Since numerous efforts have been exerted to prevent suicide by using smartphones, the movement to identify phenotypes related to suicide prevention using wearable devices is also increasing [6]. The results of these studies can be used to provide suicide-prevention interventions by optimizing the timeframe related to suicidal ideation. The amount of time they spend using suicide-related apps or the phenotype information related to suicidal thoughts and behaviors can be applied to the machine learning technique to provide specific and timely interventions. Moreover, high-risk suicide groups can be possibly monitored remotely using wearable or smart devices. Hence, active intervention could be devised in advance and remotely when the big data research reveals the most suicidal period per person.

In terms of suicide-prevention research, our study used a premade, publicly accessible Google survey platform to reduce participants’ burden. Our research team provided an online survey with a text message prompting them to complete the quick EMA survey. We also considered individual circadian rhythms for the EMA prompts, resulting in a high response rate. Using Google surveys reduces participants’ burden, including technical errors in self-produced mobile apps and privacy concerns for data security [5, 43]. Furthermore, considering that it is free to use, employing the Google survey platform was cost-effective, making it an economically viable trial in terms of EMA research. In creating a user-centered platform for clinical practice, the Google survey and a premade platform were easily adapted by individuals and were quickly distributed to society. A universal platform is recommended if it is required not only in the sector of clinical practice but also in the research sector.

#### Limitations

This study has several limitations. First, the generalizability of the current study is limited because of the small sample size and premature status of study design. However, in the field of information and communication technology research, feasibility studies with small sample sizes play a significant role in evaluating the practicality and usability of innovative interventions, which are essential for refining methodologies prior to pilot and main studies [20]. Given the increasing complexity of digital health interventions, small-sample feasibility studies help identify potential barriers and facilitators, enabling researchers to refine intervention designs and strategies while mitigating risks of failure before scaling up to larger studies [44]. Second, we did not consider the variability of the participants’ individual sleep cycles or circadian rhythms when assessing daily mood and suicidality. The personally optimized time of EMA was preset by reflecting participants’ preferences and availability for the exact time before data collection. However, given the irregular lifestyle of participants with a high risk for suicide, the survey schedule could be frequently changed. One participant requested to change the prompt time for the EMA. Thus, in future studies using EMA to investigate those with suicidal ideation in a real-world setting, such irregularity should be thoroughly considered during research design.

#### What the study adds to existing research

This feasibility study used the EMA method for the first time, with the specific designation to accommodate circadian rhythms and preferences of community-dwelling suicidal individuals in South Korea. The study presents valuable insights into the use of EMA among adults at risk of suicide and emphasizes the importance of considering mental health characteristics while implementing EMA-based research. The findings of this study can significantly contribute to the development and implementation of future suicide-prevention interventions using EMA. This study contributes to the existing literature as it confirmed that EMA research, including the use of wearable devices and intensive measurements three times a day, can be successfully implemented in adults at risk for suicide. The EMA response rate and Actiwatch adherence rate in this study were comparable to or higher than those reported in previous studies [6, 12, 32]. Furthermore, we found a positive correlation between the Actiwatch adherence rate and EMA response rate. Given the vulnerability of this population, their study adherence rate was associated with depression, anxiety, and stress. Based on these findings, mental health professionals should monitor the status of this population when their response rate decreases. Finally, the study confirmed the feasibility of conducting real-time assessments of suicide risk using a universal platform.

#### Implications for nursing practice

The use of technology in psychiatric nursing practice is continually evolving, and the EMA is a promising tool that can improve suicide risk assessment and management. Moreover, EMA can be used by mental health professionals to monitor and manage suicidal ideation in their patients, which can enhance the quality of care provided to patients. The severity of mental health symptoms in these patients is a crucial factor while considering the reliability of the EMA [44]. Patients with severe mental health problems may require additional support to fully participate in the study. Additionally, real-time event marker data collected from wearable devices can be used to predict suicidal impulse patterns, making it a valuable tool for suicide risk assessment.

By improving participant compliance, this study highlights the feasibility of EMA as a self-monitoring protocol for individuals at a distance, allowing them to actively track their symptoms. Furthermore, increasing compliance among participants can help reduce the burden on busy clinical staff responsible for monitoring responses, making EMA a more practical tool in psychiatric nursing care. This emphasizes the potential of EMA and actigraphy as effective methods for improving adherence and enhancing mental health interventions.

### Conclusion

Suicide has become a major public health issue worldwide. The study findings indicate the potential for voluntary and timely assessment of suicidal ideation, which is openly underreported in community settings. This study demonstrated a high response rate of EMA using a smartphone and a high adherence rate of wearing Actiwatch for 28 days in adults with suicidal ideation. Hence, the feasibility and participant adherence to the EMA design for assessing suicidal ideation among adults with suicide risk in South Korea are promising. Future research is needed to implement a real-time suicide-prevention approach with the mHealth app and to improve its acceptance among individuals with suicidal ideation.

## Data Availability

The data utilized and analyzed in this study can be provided by the corresponding author upon reasonable request.

## Abbreviations

App: Application
AUDIT-K: Alcohol Use Disorder Identification Test-Korean (AUDIT-K)
EMA: Ecological momentary assessment
GAD-7: Generalized Anxiety Disorder-7
ICT: Information and Communication Technology
IRB: Institutional Review Board
M: Mean
P: Probability
PHQ-9: Patient Health Questionnaire-9 (PHQ-9)
PSS: Perceived Stress Scale (PSS)
SD: Standard deviation
SPSS: Statistical Package for Social Sciences
